# Prevalence and associated factors of depression in postmenopausal women: a systematic review and meta-analysis

**DOI:** 10.1186/s12888-024-05875-0

**Published:** 2024-06-10

**Authors:** Jiaxin Li, Fangli Liu, Ziwei Liu, Mengjie Li, Yingying Wang, Yameng Shang, Yuege Li

**Affiliations:** 1 School of Nursing and Health, Henan University, Kaifeng, Henan P. R. China; 2 Institution of Nursing and Health, Henan University, Kaifeng, Henan P. R. China; 3 Xinyang Vocational and Technical College, Xinyang, Henan P. R. China; 4 The First Affiliated Hospital of Henan University of Traditional Chinese Medicine, Zhengzhou, Henan P. R. China

**Keywords:** Depression, Postmenopausal women, Meta-analysis

## Abstract

**Background:**

Depression is a prevalent mental health problem in postmenopausal women. Given its significant impact on the quality of life and overall well-being of postmenopausal women, there is need for a comprehensive review and meta-analysis of the existing research globally. This systematic review and meta-analysis evaluated the global prevalence of depression and potential associated factors in postmenopausal women.

**Methods:**

The Cochrane Library, PubMed, EMBASE, Web of Science, MEDLINE, and PsycINFO databases were systematically searched from inception to March 22, 2023. The meta-analysis used the random-effects model to calculate the prevalence of depression rates and associated factors. In addition, subgroup analysis and sensitivity analysis were performed. Publication bias was assessed using funnel plots, Egger’s test, and nonparametric trim-and-fill tests.

**Results:**

The meta-analysis included 50 studies that involved 385,092 postmenopausal women. The prevalence of depression in postmenopausal women was 28.00% (95% CI, 25.80–30.10). Among the factors relevant to depression among postmenopausal women, marital status (OR: 2.03, 95%CI: 1.33–3.11), history of mental illness (OR: 2.31, 95%CI: 1.50–3.57), chronic disease (OR: 3.13, 95%CI: 2.20–4.44), menstrual cycle (OR: 1.42, 95%CI: 1.17–1.72), abortion numbers (OR: 1.59, 95%CI: 1.40–1.80), menopausal symptoms (OR: 2.10, 95%CI: 1.52–2.90), and hormone replacement therapy (OR: 1.76, 95%CI: 1.31–2.35) were risk factors, while physical activity (OR: 0.56, 95%CI: 0.53–0.59), number of breastfed infants (OR: 0.43, 95%CI: 0.19–0.97), menopause age (OR: 0.44, 95%CI: 0.37–0.51) were preventive factors.

**Conclusions:**

This study demonstrated that the prevalence of postmenopausal depression is high, and some risk factors and protective factors associated with it have been identified. It is necessary to improve screening and management and optimize prevention and intervention strategies to reduce the harmful effects of postmenopausal depression.

**Supplementary Information:**

The online version contains supplementary material available at 10.1186/s12888-024-05875-0.

## Introduction

Depressive disorders continue to be a prominent source of global burden, with females experiencing a higher prevalence than males [1]. Women’s susceptibility to depression during reproductive events, such as premenstrual, postpartum, and menopausal transition, suggests a potential link between hormone fluctuations and depressive symptoms [2, 3]. Across their lifespan, females are at double the risk of developing depression compared to males, and the risk significantly increases after menopause [4, 5]. Menopause is defined as the absence of menstruation for 12 consecutive months [6], resulting from the depletion of the finite store of ovarian follicles, which leads to reduced secretion of estrogen and progesterone hormones [7]. Fluctuating estrogen levels can disrupt the regulation of serotonin and norepinephrine, which may contribute to depression development [8]. Postmenopause refers to the physiological period that follows menopause, commencing with the final menstrual period and extending until the end of life [7]. Depression symptoms occur during perimenopause and persist postmenopause [9]. The prevalence of depression increases in the early postmenopausal stage [10], with severe depressive mood being more common among postmenopausal women compared to those in the perimenopausal stage [11]. The manifestation of postmenopausal depression has the potential to impair functional outcomes [12], undermine quality of life [13], and diminish overall life satisfaction [10]. It is crucial to detect and treat depression early to prevent serious consequences.

Previous studies have reported the postmenopausal prevalence of depression. The prevalence of depression in postmenopausal women is 9.8%~44.7% in America [14–16], 19.6%~33.3% in China [10, 17, 18], 6.29%~28.56% in Korea [19–21], and 28.10%~46.2% in Turkey [22, 23]. Some systematic reviews have been conducted, but globally representative estimates of the prevalence of postmenopausal depression remain scarce. For instance, a meta-analysis was conducted on the prevalence of perimenopausal and postmenopausal depression in India [24], and Kruif et al. [9] compared the prevalence of depression in perimenopausal and postmenopausal women. However, their research is limited in India, or the study population is not specifically targeted at postmenopausal women. Therefore, providing a comprehensive global estimate of postmenopausal women is essential to address their mental health concerns.

Undoubtedly, postmenopausal depression is a complex condition influenced by various factors. Previous studies have summarized risk and protective factors associated with postmenopausal depression, such as marital status [20, 22, 23, 25, 26], education [20, 23, 25], economic status [20, 22], diet [19, 27, 28], health status [20, 23], and vasomotor symptoms [14, 17, 26]. However, there exist inconsistencies in the research results. Kim et al. [21] indicate that regular physical activity is a protective factor against postmenopausal depression, whereas Alam et al. [26] indicate that physical exercise is unrelated to postmenopausal depression. Therefore, it is crucial to synthesize diverse research results to conduct a comprehensive analysis to clarify the strength of these associations.

Understanding the burden and related factors of postmenopausal depression can help in developing better screening, management, prevention, and intervention strategies for postmenopausal women. Considering the gaps in current research regarding depression in postmenopausal women, we performed a systematic review and meta-analysis to calculate the prevalence of postmenopausal depression globally and estimate associated factors.

## Methods

The systematic review and meta-analysis followed the checklist outlined by Preferred Reporting Items for Systematic Reviews and Meta-Analyses 2020 (PRISMA 2020) guidance [29] (Additional file 1) and was registered in the PROSPERO database (ID: CRD42023410004).

### Search strategy

Utilizing the PEO framework, which was modified from PICO questions, to clearly define the review inquiry. Population: Postmenopausal women; Exposure: Factors associated with depression among postmenopausal women; Outcome: Prevalence of depression among postmenopausal women.

Literature searches were performed in the Cochrane Library, PubMed, EMBASE, Web of Science, MEDLINE, and PsycINFO databases from inception to March 22, 2023, for relevant studies reported on postmenopausal depression. In this study, self-reported screening instruments or diagnoses by a physician were used to identify depression. The search focused on the prevalence of postmenopausal depression and the related factors. The search strategy detailed is shown in Additional file 2. To identify additional studies, the reference lists of articles retrieved were also reviewed. Articles were limited to being published in English.

### Inclusion and exclusion criteria

The inclusion criteria of studies included the following: (1) cross-sectional or cohort studies; (2) study population is postmenopausal women; (3) depression was evaluated by a validated self-report screening instrument or diagnosed by a physician; and (4) studies have reported the effect estimates with the prevalence of postmenopausal depression or 95% confidence intervals (CI) of the associated factors. Exclusion criteria of studies included the following: (1) Study population conducted not only on postmenopausal women (i.e., conducted combined with menopausal women or perimenopausal, etc. ); (2) Studies did not provide enough information to calculate effect estimates. (3) Reviews, quasi-experimental, randomized controlled trials and case-control studies, essays, conference abstracts, letters, and commentaries.

### Study selection

Two review authors (ZWL and YYW) conducted the systematic literature search independently and exported all retrieved articles to the EndNote 20 reference manager software to eliminate duplicates and facilitate the screening process. Subsequently, screened titles and abstracts, followed by reading full texts to identify eligible studies based on predefined inclusion and exclusion criteria. Any disagreements encountered during this process were resolved through discussions led by the third investigator (JXL) until a consensus was achieved.

### Data extraction

Microsoft Excel spreadsheet (2016) was utilized for data extraction. Two authors (YMS and YGL) performed data extraction independently on key information: the first author’s name, publication year, study design, country, survey year, sampling, population type, sample size, mean age of samples, measure of depression, cutoff points of instruments, prevalence, associated factors, and effect sizes of the odds ratio (OR) with 95% CI. Disagreements were resolved through discussions by the third author (FLL) until a consensus was reached.

### Quality assessment

Quality assessments were performed by two reviewers (ZWL and MJL) independently. Disagreements were resolved through discussions by the third author (FLL) until a consensus was reached. To evaluate the quality of cross-sectional studies, the checklist involving eleven items recommended by the Agency for Healthcare Research and Quality (AHRQ) was used [30]. The score range is 0–11. Scores 0–3 are assigned to low-quality studies, 4–7 to moderate-quality studies, and 8–11 to high-quality studies. The Newcastle‒Ottawa Scale (NOS) [31] was used to evaluate the quality of cohort studies, including a selection of study groups (4 points); 2) comparability of groups (2 points); and ascertainment of exposure and outcomes (3 points), which ranges from 0 to 9. The studies scores 0–3 are assigned to low quality, 4–6 are assigned to moderate quality, and 7–9 are assigned to high quality [32].

### Data analysis

Stata Statistical software version 15.1 was utilized for data analysis. First, to estimate the postmenopausal prevalence of depression, the effect sizes to be used are prevalence and 95% CI. The meta-analysis used the random-effects model to calculate the prevalence of depression rates, accounting for heterogeneity between studies. When several studies utilize the same scale but define the depression prevalence using different cut-off values, we select the cut-off values used in the original studies to calculate the depression rates for estimating the prevalence. Subgroup analyses were undertaken to explore potential heterogeneity across studies. Differences among subgroups may be tested using meta-regression analysis, which was based on random-effect analysis, and differed significantly according to the economic status of countries, study type, sample size, year of publication, and assessment instrument (all *p* values < 0.05). Heterogeneity will be assessed with the Cochran Q and I² tests. Heterogeneity will be considered low, moderate, and high if I² is less than 25%, between 25% and 50%, and greater than 50%, respectively. Second, to estimate the factors related to postmenopausal depression, the effect sizes to be used are each potential factor OR and 95% CI, and meta-analysis used the random-effects model. We consider a variable as a possible factor linked to depression in postmenopausal women only if at least two studies provide its OR and 95% CI. If reported adjusted for multivariable analyses, we extracted the adjusted OR value. Potential bias was examined using the funnel plot, Egger test, and the nonparametric trim-and-fill method. In sensitivity analyses, a series of systematic exclusions were conducted, removing one study at a time from the overall data to observe changes in the overall prevalence estimate and identify any potential studies that may have a disproportionate impact on the estimated prevalence of postmenopausal depression. All tests were two-tailed, with a *p* value < 0.05 indicating statistical significance.

## Results

### Searching results

**Fig. 1:**
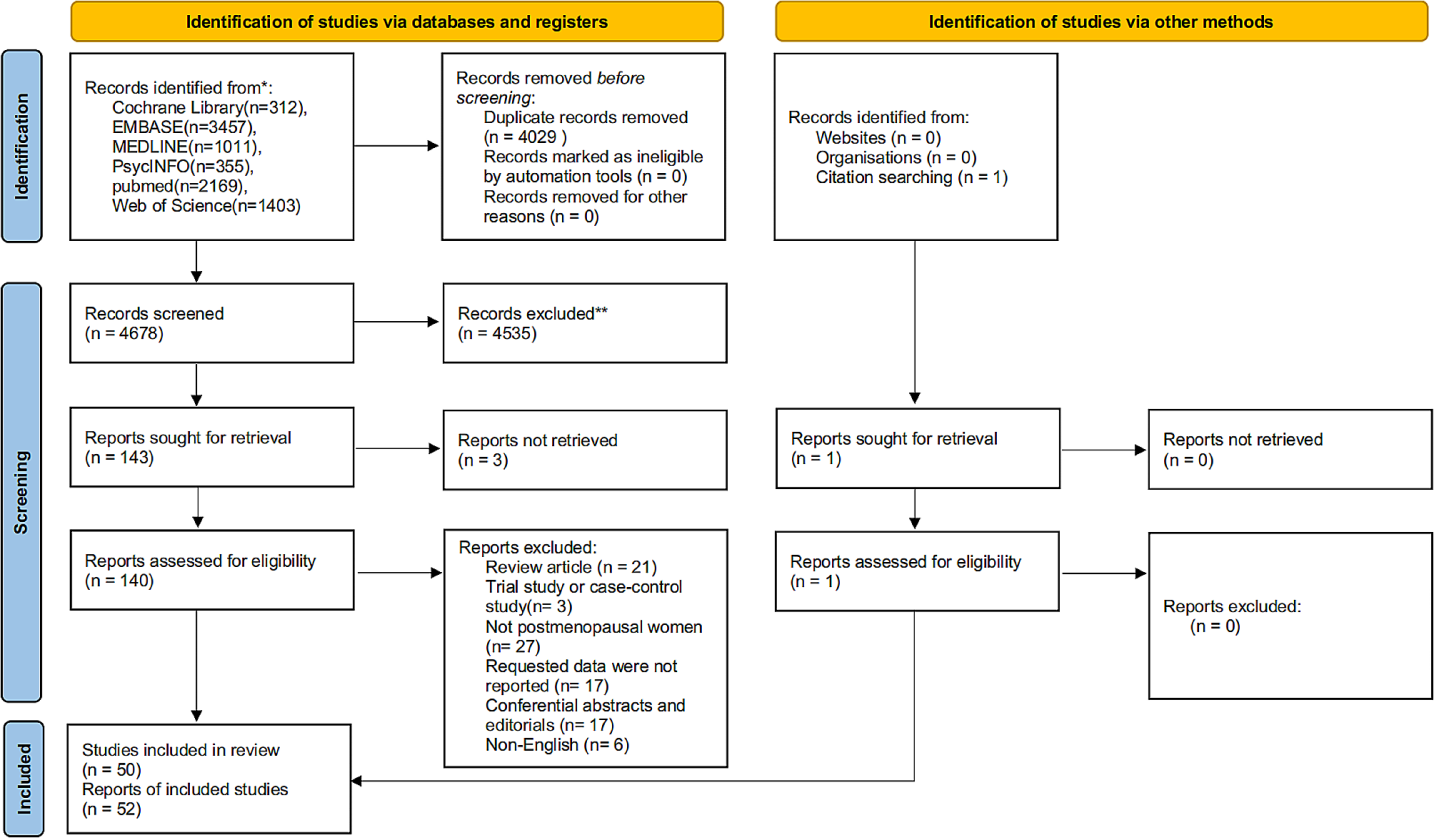
PRISMA flow diagram of search results

In total, 8,708 records were found in the database, and after removing duplicates, 4,678 records remained. Depending on the inclusion and exclusion criteria, 49 studies were included after reading the title, abstract, and full text. Additionally, 1 study was manually added by searching the references of retrieved studies. Finally, the systematic review and meta-analysis included a total of 52 reports from 50 studies. The flow diagram of search results is shown in Fig. 1.

### Study characteristics

Fifty studies [33–82]provided prevalence ratios. Among them, 22 studies [35, 37, 43, 46–48, 52, 57–59, 62, 63, 67, 68, 72, 76–82] provided odds ratios between the factors and depression among postmenopausal women. In the meta-analysis, 44 studies [33–76] were cross-sectional, and 6 studies [77–82] were cohort studies. Notably, the study by Ina et al. [36] separately reported the prevalence of postmenopausal depression in Korea, China, and Japan, and therefore these data were analyzed as three distinct reports.

Specifically, 52 reports from 50 studies were included, with eight were conducted in China [36, 52, 53, 59, 62, 69, 70, 76], seven in the USA [33, 34, 46, 75, 77, 79, 82], seven in Korea [36, 47, 48, 55, 57, 58, 67], six in Poland [38, 40, 44, 50, 51, 66], six in Iran [45, 49, 54, 64, 65, 68], five in India [41, 56, 60, 61, 71], four in Turkey [35, 37, 63, 73], two in Australia [39, 78], two in France [80, 81], one in Japan [36], one in Spain [42], one in Greece [72], one in Jordan [74], and one in several Mediterranean islands [43]. The measurement of depression was evaluated by a validated self-report screening instrument or diagnosed by a physician. Specifically, 14 used the Beck Depressive Inventory (BDI), 12 used the Center for Epidemiologic Studies Depression Scale (CES-D), 5 used the Hamilton Depression Scale (HAM-D), 4 used the Geriatric Depression Scale (GDS), 3 used the 9-item Patient Health Questionnaire (PHQ-9), 3 used the Zung’s Self-rating Depression Scale (SDS), 7 used the Others (6-items CES-D/Diagnostic Interview Schedule, 8-item CES-D/Diagnostic Interview Schedule, 6 items CES-D, Burnam 8-item scale, Depression Anxiety Stress Scale 21, Inventory of Depressive Symptomatology-Self Report scale, The hospital anxiety and depression scale) each used in one study, and 4 used diagnosed by a physician. Studies were published between 1999 and 2023, and the sample size ranged from 100 to 93,676, totaling 385,092. Detailed information on the included studies (Additional file 3).

### Quality assessment

Of all studies,11 studies [39, 47, 52, 53, 59, 62, 76, 78–80, 82]scored high quality, 30 studies [33, 34, 37, 38, 43–46, 48, 49, 51, 54–58, 60, 63–65, 67–74, 77, 81]had moderate quality, and 9 studies [35, 36, 40–42, 50, 61, 66, 75]had low quality. The quality score (Additional file 4) is presented in the additional file.

### The prevalence of depression in postmenopausal women

**Fig. 2:**
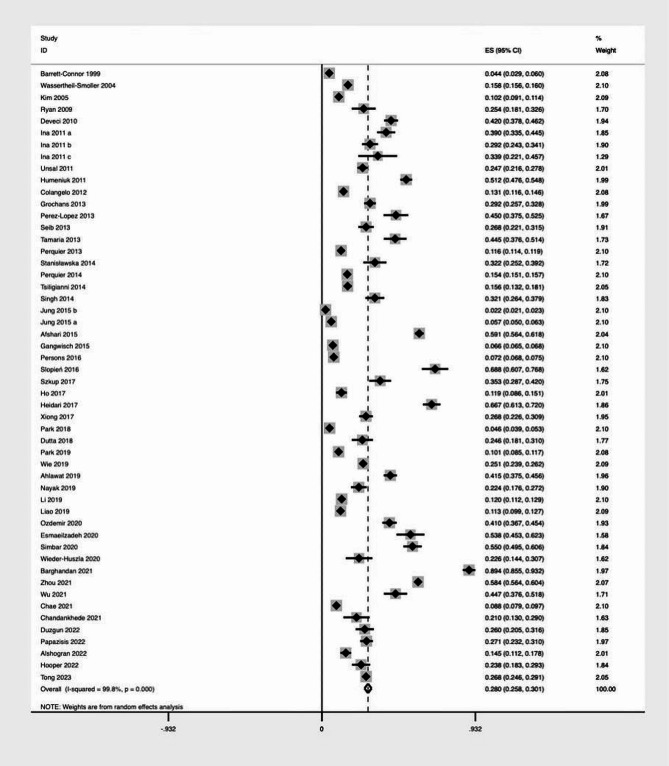
Forest plot for the prevalence of depression

In the meta-analysis, 50 studies including 52 reports showed that the prevalence of postmenopausal depression was 28.00% (95% CI, 25.80-30.10%, I^2^ = 98.8%, *p* value < 0.001) (Fig. 2).

### Subgroup analysis of the prevalence of depression in postmenopausal women

According to the 52 reports on the prevalence of postmenopausal depression, the pooled prevalence of depression in developing countries [35–38, 40, 41, 44, 45, 49–54, 56, 59–66, 68–71, 73, 74, 76] (37.30%, 95% CI: 29.60–44.90) is higher than that in developed countries [33, 34, 36, 39, 42, 43, 46–48, 55, 57, 58, 67, 72, 75, 77–82] (15.90%, 95% CI: 13.30–18.60). In addition, in cohort studies [77–82], the prevalence of depression (13.80%, 95% CI: 10.60–17.00) was lower than that in cross-sectional studies [33–76] (30.00%, 95% CI: 27.50–32.40). Studies that had a sample size of below 1000 [33, 35–45, 50–54, 56, 60, 61, 63–66, 68, 70–75, 78] (35.00%, 95% CI: 28.00–42.00) recorded a higher prevalence than those that had a sample size greater than or equal to 1000 [34, 46–49, 55, 57–59, 62, 67, 69, 76, 77, 79–82] (16.80%, 95% CI: 13.60–19.90).

Moreover, the prevalence of depression was higher in studies published in 2016 or later [50–76, 82] (31.20%, 95% CI: 26.40–36.00) than in those published before 2016 [33–49, 77–81] (24.70%, 95% CI: 21.80–27.60). Last, the highest prevalence of depression was reported for studies that used the HAM-D instrument (45.20%, 95% CI: 31.00-59.40), followed by studies that used the BDI instrument (38.10%, 95% CI: 24.40–51.90) and studies that used the GDS instrument (29.10%, 95% CI: 16.70–41.50) (Table 1).

**Table 1:** Subgroup analyses of the prevalence of depression in postmenopausal women

Subgroups	Categories (number of reports)	Prevalence (%)	95%CI (%)	I^2^ (%)	*p* value for hetergeneity	*p* value between groups
Economic status of countries	developed countries (22)	15.90	13.30–18.60	99.9	<0.001	<0.001
	developing countries (30)	37.30	29.60–44.90	99.4	<0.001	
Study type	cross-sectional (46)	30.00	27.50–32.40	99.7	<0.001	<0.001
	cohort (6)	13.80	10.60–17.00	99.7	<0.001	
Sample size	<1000 (34)	35.00	28.00–42.00	98.9	<0.001	<0.001
	≥ 1000 (18)	16.80	13.60–19.90	99.9	<0.001	
Year of publication	<2016 (24)	24.70	21.80–27.60	99.9	<0.001	<0.001
	≥ 2016 (28)	31.20	26.40–36.00	99.6	<0.001	
Instrument	BDI (14)	38.10	24.40–51.90	99.4	<0.001	<0.001
	CES-D (12)	26.70	23.50–29.80	99.6	<0.001	
	GDS (4)	29.10	16.70–41.50	96.0	<0.001	
	HAM-D (5)	45.20	31.00-59.40	97.8	<0.001	
	SDS (3)	19.50	7.90–31.10	96.2	<0.001	
	PHQ-9 (3)	16.30	8.50–24.00	98.8	<0.001	
	diagnosed by a physician (4)	11.00	4.00-18.10	99.7	<0.001	
	Other (7)	15.60	12.8–18.4	99.8	<0.001	

BDI, Beck Depressive Inventory; CES-D, Center for Epidemiologic Studies Depression Scale; GDS, Geriatric Depression Scale; HAM-D, Hamilton Depression Scale; SDS, Zung’s Self-rating Depression Scale; PHQ-9,9-item Patient Health Questionnaire; CI, confidence interval

### Factors associated with depression among postmenopausal women

These risk factors, identified in the included 22 studies, can be categorized as follows demographic characteristics, lifestyle factors, medical and health factors, and reproductive health factors. For detailed information refer to Additional File 5.

### Demographic characteristics

For this part, age, marital status, number of children, education, and working status were associated with depression among postmenopausal women.

Marital status with three reports can be used for the quantitative meta-analysis. Compared with married, postmenopausal women who are single, widowed, or divorced were significantly more susceptible to depression (OR: 2.03, 95%CI: 1.33–3.11) (Table 2).

### Lifestyle factors

Lifestyle factors associated with depression among postmenopausal women include weight, dietary pattern, physical activity, alcohol consumption, and smoking status.

Two reports provided data on physical activity used for meta-analysis, physical activity reduces the risk of depression among postmenopausal women, compared to those without physical exercise or with irregular physical exercise (OR: 0.56, 95%CI: 0.53–0.59) (Table 2).

### Medical and health factors

For this part, the history of mental illness, the experience of violence, life events, chronic disease, disability, and cardiovascular disease events were associated with depression among postmenopausal women.

Three reports and two reports provided data on the factors of history of mental illness and chronic disease, respectively, that can be used for meta-analysis. The results showed that postmenopausal women with a history of mental illness (OR: 2.31, 95%CI: 1.50–3.57) and chronic disease (OR: 3.13, 95%CI: 2.20–4.44) were more prone to depression (Table 2).

### Reproductive health factors

Reproductive health factors include menstrual cycle, menstrual cycle length, reproductive period, full-term pregnancies, experienced pregnancy, abortion numbers, number of induced abortions, number of breastfed infants, periods of breastfed infants, contraceptive surgery, menopause age, type of menopause, oral contraceptive usage, hormone replacement therapy, sex hormones, and menopausal symptoms were associated with depression among postmenopausal women.

Among these factors, menstrual cycle, abortion numbers, number of breastfed infants, menopause age, menopausal symptoms, and hormone replacement therapy provided data can be used for the quantitative meta-analysis. Specifically, menstrual cycle irregular (OR: 1.42, 95%CI: 1.17–1.72), abortion numbers greater than 0 (OR: 1.59, 95%CI: 1.40–1.80), experiencing menopausal symptoms (OR: 2.10, 95%CI: 1.52–2.90), used hormone replacement therapy (OR: 1.76, 95%CI: 1.31–2.35) were more prone to postmenopausal depression. Number of breastfed infants greater than 1(OR: 0.43, 95%CI: 0.19–0.97), menopause age later (OR: 0.44, 95%CI: 0.37–0.51) are less likely to suffer from postmenopausal depression (Table 2).

**Table 2:** Meta-analyses of factors associated with depression symptoms

Factors	No. of reporters	Min OR	Max OR	Pooled OR with 95%CI	I^2^(%)	*p* value for heterogeneity
Marital status (reference: married)	3	1.65	3.48	2.03 (1.33–3.11)	54.5	0.111
Physical activity (reference: none/no regular)	2	0.56	0.62	0.56 (0.53–0.59)	0.0	0.647
History of mental illness (reference: no)	3	1.65	3.58	2.31 (1.50–3.57)	58.5	0.090
Chronic disease (reference: no)	2	2.80	3.58	3.13(2.20–4.44)	0.0	0.494
Menstrual cycle (reference: regular)	2	1.35	1.73	1.42(1.17–1.72)	25.8	0.246
Menstrual cycle length (reference: shorter)	2	1.05	1.15	1.08(1.00-1.18)	62.7	0.102
Reproductive period (reference: shorter)	4	0.41	0.97	0.60(0.36–1.01)	97.1	<0.001
Abortion numbers (reference: 0)	3	1.45	1.74	1.59(1.40–1.80)	0.0	0.701
Number of breastfed infants (reference: 0–1)	3	0.23	0.81	0.43(0.19–0.97)	84.8	0.001
Menopause age (reference: ≤46)	2	0.35	0.45	0.44(0.37–0.51)	2.1	0.312
Type of menopause (reference: artificial)	2	1.15	1.61	1.36(0.98–1.89)	93.8	<0.001
Menopausal symptoms (reference: no)	3	1.71	3.65	2.10(1.52–2.90)	69.6	0.037
Oral contraceptive usage (reference: never)	2	1.15	1.55	1.21(0.97–1.49)	22.7	0.255
Hormone replacement therapy (reference: never)	6	1.07	2.89	1.76(1.31–2.35)	93.7	<0.001

CI, confidence interval; OR, odds ratio

### Publication bias

**Fig. 3:**
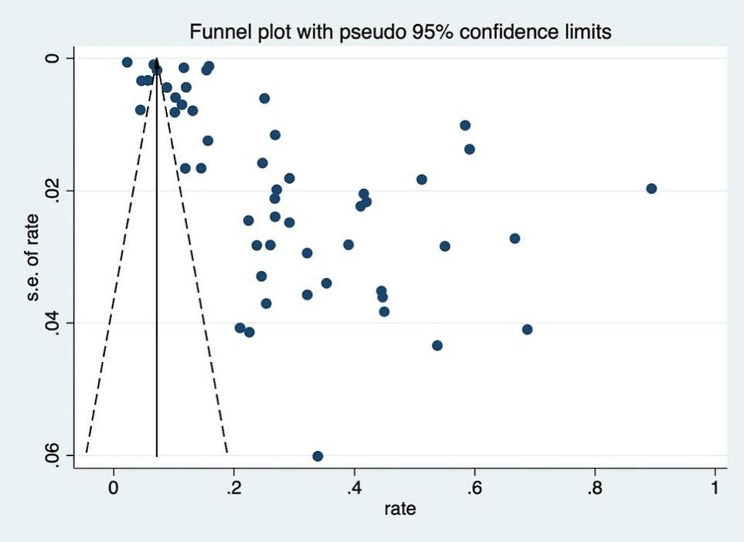
Funnel plot for publication bias for depression

The analysis of publication bias revealed that there might be some asymmetry in the funnel plot (Fig. 3). Egger’s test indicated evidence of publication bias (*p* value<0.001). The corrected OR using the trim-and-fill method was 31.17% (95% CI, 24.40–39.10; random-effects model, *p* value < 0.001).

### Sensitivity analysis

**Fig. 4:**
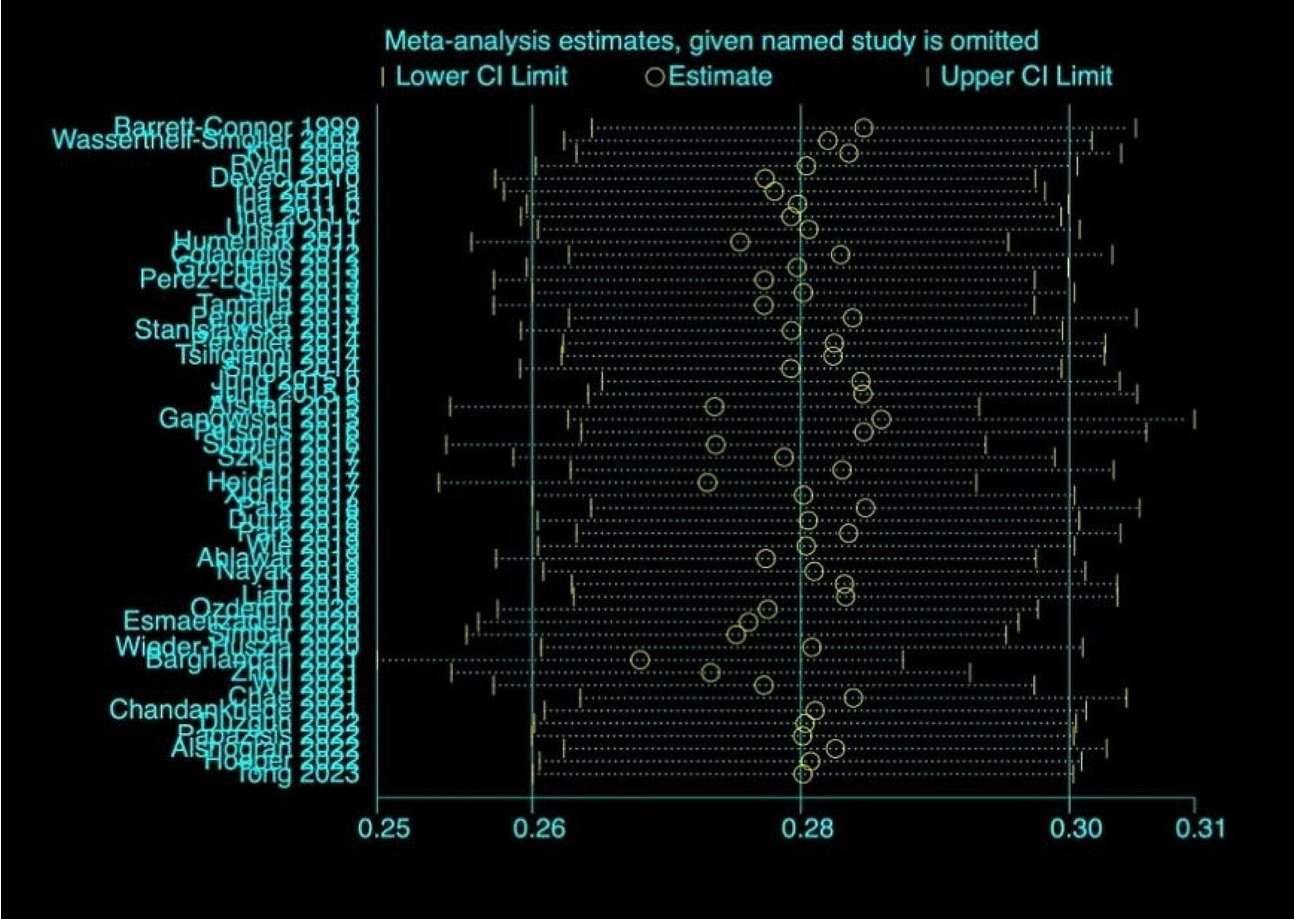
Sensitivity analysis for the prevalence of depression

The sensitivity analysis showed that the summary of risk estimates would not be significantly impacted by any single influential study (Fig. 4). The prevalence of depression remained unchanged after removing a single study.

## Discussion

The present meta-analysis showed that the prevalence of depression among postmenopausal women was 28.0%, emphasizing the importance of paying attention to their mental health. Ten factors were associated with depression symptoms, including marital status, physical activity, history of mental illness, chronic disease, menstrual cycle, abortion numbers, number of breastfed infants, menopause age, menopausal symptoms, and hormone replacement therapy were related with depression among postmenopausal women. Therefore, hospital and community health workers should consider the related factors when developing strategies to prevent and intervene in depression in postmenopausal women.

According to this meta-analysis, the pooled prevalence of postmenopausal depression worldwide was 28.0% (95% CI, 25.8–30.1), which was lower than in previous meta-analysis studies. A meta-analysis study on 6,389 menopausal Chinese women with 23 cross-sectional studies in which the prevalence of depression was 36.3% (95% CI: 27.5–45.1) [83]. Similarly, a meta-analysis study in subgroup analyses with 7 articles reported on postmenopausal women in India in which the prevalence of depression was 37.83% (95% CI: 25.83–51.53) [24]. The pooled prevalence of postmenopausal depression differences in these meta-analyses might be related to the variations in the study population and diversity in economic status between studies included. This meta-analysis included a broader range of studies, on the economic status of countries both developing and developed, whereas previous reviews were conducted exclusively in developing countries. Additionally, subgroup analysis confirmed that the prevalence of postmenopausal depression is lower in developed countries.

In the subgroup analysis, the prevalence of depression in postmenopausal women in developing countries was higher than that in developed countries. The prevalence of postmenopausal depression in India (37.83%) was similar to that reported for developing countries (37.30%) in this study [24]. The significant regional variation in the prevalence of depression could be attributed to the gap in medical resources and healthcare coverage access. In this meta-analysis, studies with small sample sizes showed higher prevalence rates than larger ones. Generally, studies with larger sample sizes are more reliable and could be better generalized to the target population [84]. There was also a significant discrepancy between cross-sectional and cohort studies. This could be because this study conducted a large number of cross-sectional studies with many participants and relatively fewer cohort studies. Therefore, this finding suggests selection bias in sample size and the study design that should be considered in future studies. Additionally, the prevalence of postmenopausal depression was higher in studies published after 2016 than in those published before. This may be caused by modernity, which has gradually increased lifestyle and psychosocial issues, such as more social isolation and less intimate engagement with their families [85]. Additionally, midlife women are more susceptible to stress and encounter a greater number of stressors, which can lead to stress-induced depression [86, 87]. It has been observed that the prevalence of depression assessed by self-reported screening instruments is higher compared to the diagnosis by a physician. One reason for this could be that the ‘gold standard’ of diagnosis by a physician uses more stringent criteria of structured clinical interviews than self-report instruments [88]. In this study, most studies relied on self-report instruments to assess depression, and different instruments contributed to several differences. While validated diagnostic interviews require more time and resources, self-reported screening instruments with high clinical utility may overestimate the actual prevalence of depression [89]. There is no specific scale for postmenopausal depression, and several generally validated screening measures may be used for categorical determination of depression. It is necessary to explore which scale is most appropriate.

Previous meta-analysis has confirmed that a later age at menopause and a longer reproductive period are associated with a reduced risk of depression among postmenopausal women [90]. Evidence suggests that Hormone Replacement Therapy can improve depressive symptoms and can be considered for use in perimenopausal women without contraindications to alleviate and manage depressive symptoms [91–93]. However, our study found that prior use of Hormone Replacement Therapy may increase the risk of postmenopausal depression. Furthermore, our study further clarified the relationship between various factors and postmenopausal depression.

In the demographic characteristics factors, marital status with single, widowed, or divorced is a risk factor for postmenopausal depression. Studies have shown that midlife women who receive high social support have a 20% lower risk of developing depression [94]. In addition, adults tend to rely more on their spouses as a source of support, which is a consistent protective factor against depression [95]. Therefore, psychological care should be strengthened for postmenopausal women, especially those who are single, divorced, or widowed.

In lifestyle factors, physical activity is a preventive factor. A systematic review revealed that physical activity improves postmenopausal women’s physical and mental well-being [96], and guidelines indicated that exercise can be recommended for women with depression after menopause [91]. However, further research is needed to determine the most beneficial forms, frequencies, and intensities of exercise for postmenopausal depression.

In medical and health factors, history of mental illness and chronic diseases are risk factors. A history of mental illness, specifically depressive episodes, is the strongest predictor for depression during women’s midlife years [97]. This relationship between a history of mental illness and depression among women in other population groups has been confirmed [98–100]. A meta‑analysis revealed that physical illness was strongly associated with depression in the elderly [84]. Postmenopausal women are at increased risk for chronic disease, resulting in low functioning, pain or discomfort, and disability, which may affect the development of depression symptoms [101]. Hence, Screening and management should be undertaken in patients with previous depressive disorders and chronic disease promptly to prevent postmenopausal depression.

In reproductive health factors, menstrual cycle, abortion numbers, menopausal symptoms, and hormone replacement therapy were risk factors, and the number of breastfed infants and menopause age were preventive factors. This meta-analysis found that a regular menstrual cycle in premenopause decreased the risk of depressive symptoms after menopause, regardless of the cycle length. Previous studies have found that irregular cycle variability has also been revealed to be significantly associated with antenatal depression [102]. The association between the menstrual cycle and depression could be supported by a possible biological mechanism, the hypothalamic‒pituitary‒adrenal axis, which is implicated in playing a role in regulating mood and responding to stress [103–105]. The number of total abortions is a contributing factor of postmenopausal depression. Women who have recurrent pregnancy loss are at a greater risk of suffering from moderate or severe depression [106]. An increased number of spontaneous and induced abortions is related to an increased likelihood of depression [48, 58]. The longer a woman breastfeeds during reproductive years, the greater the association with lifelong health benefits [107]. In this study, postmenopausal depression decreased as the number of breastfed infants increased, perhaps because of increased social support from having multiple children [95]. The study found that for each additional year of estrogen exposure, which is the time between menarche and the onset of menopause, the odds of postmenopausal depression decrease by 15% [108]. Similarly, a meta-analysis revealed an inverse relationship between later age at menopause and depression [90]. Vasomotor symptoms can persist for up to ten years or more [109], and severe vasomotor symptoms may increase the risk of postmenopausal depression [110]. Estrogen therapy can be beneficial in treating depression for some perimenopausal women [91]. However, there may be an increased risk for adverse events and side effects in postmenopausal women [109]. A meta-analysis indicated no clinically significant effect of bioidentical estrogen on depressive symptoms [111]. Therefore, the potential for adverse effects on postmenopausal women should be considered when using hormone replacement therapy in the future.

### Strength and limitations

This study has contributed to the evidence regarding the global prevalence of postmenopausal depression and its associated factors. It provides a reference for future healthcare professionals in preventing and reducing postmenopausal depression. Furthermore, a systematic and comprehensive search was conducted across multiple databases, and a random-effects model and subgroup analysis were employed to explain significant differences in heterogeneity among studies.

However, some limitations in this meta-analysis should be considered. First, a high degree of heterogeneity was observed in this meta-analysis, although subgroup analyses were conducted. Meta-analysis of epidemiological studies is inevitably associated with high heterogeneity, which may also result from differences in the study design, sampling methods, cultural contexts, measurements, and cutoff points. Second, most of the studies the meta-analysis included identified depression by self-report instruments, which may have overestimated the prevalence. Third, in this meta-analysis, the studies included are limited to those published in English. Most study designs were cross-sectional, and sample sizes were relatively small. Fourth, Among the meta-analyses of influencing factors, most only included a small number of studies, and subgroup analyses were not performed, which may not fully reflect various complex situations and factors. These could potentially have an impact on the generalizability of the research findings. Finally, this study found the existence of publication bias, which may render the conclusions less accurate and reliable. Therefore, the findings should be treated with caution.

## Conclusion

In summary, this study demonstrates that the incidence of depression is high among postmenopausal women. Ten factors were confirmed to be related to depression in postmenopausal women, including marital status, physical activity, history of mental illness, chronic disease, menstrual cycle, abortion number, number of breastfed infants, menopause age, menopausal symptoms, and hormone replacement therapy. This suggests that we should attach importance to the mental health of postmenopausal women and promptly provide regular screening and identification for depression. Associated factors could also be used for early assessment and detection of higher risk, a personalized approach, and a suitable follow-up schedule for depression in postmenopausal women to reduce the negative effects of postmenopausal depression.

### Electronic supplementary material

Below is the link to the electronic supplementary material.

Supplementary Material 1: PRISMA checklist

Supplementary Material 2: Search strategy

Supplementary Material 3: Characteristics of studies included in the metanalysis of the prevalence of depression symptoms among postmenopausal women 

Supplementary Material 4: Quality assessment of included studies

Supplementary Material 5: Included studies of factors associated with depression symptoms 

## Data Availability

The datasets supporting the conclusions of this article are included within the article.

## Abbreviations

CI: Confidence interval
OR: Odds ratio
AHRQ: Agency for Healthcare Research and Quality
NOS: Newcastle‒Ottawa Scale
BDI: Beck Depressive Inventory
CES: D-Center for Epidemiologic Studies Depression Scale
GDS: Geriatric Depression Scale
HAM: D-Hamilton Depression Scale
SDS: Zung’s Self-rating Depression Scale
PHQ: 9-9-item Patient Health Questionnaire
