# Supplementary material for: Prevalence and associated factors of depression in postmenopausal women: a systematic review and meta-analysis
Source: BMC Psychiatry. 2024 Jun 10;24:431. doi: 10.1186/s12888-024-05875-0 (PMC11165857; doi:10.1186/s12888-024-05875-0)
Supplement: Supplementary file 2 — Supplementary Material 2: Search strategy [file 12888_2024_5875_MOESM2_ESM.docx]

**Additional file 2:**

**Search terms in PubMed, EMBASE, Web of Science, MEDLINE, and PsycINFO databases are included below. All searches were conducted on March 22, 2023.**

| **Databases** | **Search terms** | **Number of records** |
| --- | --- | --- |
| **PubMed** | ("depressed"[All Fields] OR "depression"[MeSH Terms] OR "depression"[All Fields] OR "depressions"[All Fields] OR "depression s"[All Fields] OR "Depressive Disorder"[MeSH Terms] OR ("depressive"[All Fields] AND "disorder"[All Fields]) OR "Depressive Disorder"[All Fields] OR "depressivity"[All Fields] OR "depressive"[All Fields] OR "depressively"[All Fields] OR "depressiveness"[All Fields] OR "depressives"[All Fields] OR "Depressive Disorder"[All Fields] OR "depressive symptom*"[All Fields] OR "Emotional Depression"[All Fields] OR "depressive disorder*"[All Fields] OR "depressive neuro*"[All Fields] OR "melancholia*"[All Fields] OR "depress*"[All Fields]) AND ("postmenopausal"[All Fields] OR "postmenopausally"[All Fields] OR "postmenopausals"[All Fields] OR "postmenopause"[MeSH Terms] OR "postmenopause"[All Fields] OR "postmenopausic"[All Fields] OR "postmenopausal period"[All Fields] OR "post menopause*"[All Fields] OR "Post-menopausal Period"[All Fields] OR "post menopause*"[All Fields] OR "Post-menopausal Period"[All Fields]) | **2,169 results** |
| **EMBASE** | #1 'depression'/exp OR depression  #2 'depressive disorder' OR 'depressive symptom*' OR 'emotional depression' OR ' depressive disorder*' OR 'depressive neuro* ' OR 'melancholia*' OR 'depress*'  #3 #1 OR #2  #4 'postmenopause' OR ' postmenopausal period' OR 'post-menopause* ' OR 'post menopause*' OR 'post-menopause period ' OR 'post menopausal period'  #5 #3 AND #4 | **3,457 results** |
| **Cochrane Library** | #1 MeSH descriptor: [Depression] explode all trees  #2 MeSH descriptor: [Depressive Disorder] explode all trees  #3 ("Depressive Symptom*") OR ("Emotional Depression") OR ("Depressive Disorder*") OR ("Depressive Neuro*") OR (Melancholia*) OR (Depress*)  #4 #1 OR #2 OR #3  #5 MeSH descriptor: [Postmenopause] explode all trees  #6 ("Postmenopausal Period") OR ("Post-Menopause*") OR ("Post Menopause*") OR ("Post-Menopause Period") OR ("Post Menopause Period")  #7 #5 OR #6  #8 #4 AND #7 | **312 results** |
| **Web of Science** | #1 (((((((TS=(Depression)) OR TS=(“Depressive Disorder”)) OR TS=(“Depressive Symptom*”)) OR TS=(“Emotional Depression”))OR TS=(“Depressive Disorder*”))OR TS=(“Depressive Neuro*”)) OR TS=(Melancholia*)) OR TS=(Depress*)  #2 (((((TS=(Postmenopause)) OR TS=(“Postmenopausal Period”)) OR TS=(“Post-Menopause*”)) OR TS=(“Post Menopause*”)) OR TS=(“Post-menopausal Period”))OR TS=(“Post menopausal Period”)  #3 #1 AND #2 | **1,402 results** |
| **Medline** (**via EBSCOhost)** | S1 Depression OR "Depressive Disorder" OR "Depressive Symptom*" OR "Emotional Depression" OR "Depressive Disorder*" OR "Depressive Neuro*" OR Melancholia* OR Depress*  S2 Postmenopause OR "Postmenopausal Period" OR "Post-Menopause*" OR "Post Menopause*" OR "Post-Menopause Period" OR "Post Menopause Period"  S3 S1 AND S2 | **1,011 results** |
| **psycINFO (via Ovid)** | #1 Depression.mp. OR Depressive Disorder.mp. OR Depressive Symptom*.mp. OR Emotional Depression.mp. OR Depressive Disorder*.mp. OR Depressive Neuro*.mp. OR Melancholia*.mp. OR Depress*.mp.  #2 Postmenopause.mp. OR Postmenopausal Period.mp. OR Post-Menopause*.mp. OR Post Menopause*.mp. OR Post-Menopause Period.mp. OR Post Menopause Period.mp.  #3 #1 AND #2 | **355 results** |
