# Supplementary material for: Prevalence and associated factors of depression in postmenopausal women: a systematic review and meta-analysis
Source: BMC Psychiatry. 2024 Jun 10;24:431. doi: 10.1186/s12888-024-05875-0 (PMC11165857; doi:10.1186/s12888-024-05875-0)
Supplement: Supplementary file 3 — Supplementary Material 3: Characteristics of studies included in the metanalysis of the prevalence of depression symptoms among postmenopausal women [file 12888_2024_5875_MOESM3_ESM.docx]

**Additional file 3:**

**Table 1 Characteristics of studies included in the meta-analysis of the prevalence of depression symptoms among postmenopausal women**

| Author（year） | Country | Study design | Year of survey | Sampling | Population type | Age(years) | Sample size | Prevalence (%) | Tools | cutoff point | Quality  score |
| --- | --- | --- | --- | --- | --- | --- | --- | --- | --- | --- | --- |
| Barrett-Connor (1999)^[1]^ | America | cross-sectional | 1984-1987 | NR | postmenopausal women | median=75 | 699 | 4.43 | BDI | ≥13 | 6 |
| Kim (2005)^[2]^ | America | cross-sectional | 1998.03-2001.08 | NR | postmenopausal women | range:50-83 | 2627 | 10.24 | CES-D (6-items) / DIS | ≥0.06 | 4 |
| Deveci (2010)^[3]^ | Turkey | cross-sectional | NR | randomly sampling | postmenopausal women | mean=57.08  SD=6.76 | 519 | 42.20 | BDI | ＞17 | 3 |
| Ina (2011)^[4]^ | Korea, China, Japan | cross-sectional | NR | NR | postmenopausal women | Korea: mean=59.1  SD=5.9  China: mean=59.7  SD=5.5  Japan: mean=58.9  SD=11.6 | 698 | Korea: 39.0%  China: 29.2%  Japan: 33.9% | GDS | mild:6-9  severe: ≥10 | 2 |
| Unsal (2011)^[5]^ | Turkey | cross-sectional | 2009.05-06 | NR | postmenopausal women | mean=56.64  SD=6.38  range:45-65 | 744 | 24.73 | BDI | ＞17 | 6 |
| Humeniuk (2011)^[6]^ | Poland | cross-sectional | 2011 | randomly sampling | postmenopausal women | range:52-60 | 746 | 51.21 | BDI | mild:10-19  moderate:20-25  severe: ≥26 | 7 |
| Seib (2013)^[7]^ | Australia | cross-sectional | 2011 | NR | postmenopausal women | mean=64.8  SD=2.8  range:60-70 | 343 | 26.82 | CES-D | ≥16 | 8 |
| Grochans (2013)^[8]^ | Poland | cross-sectional | NR | NR | postmenopausal women | mean=57.5  SD=6.4 | 630 | 29.21 | BDI | ≥10 | 3 |
| Tamaria (2013)^[9]^ | India | cross-sectional | NR | NR | postmenopausal women | range: > 40  mean=57.9  SD=9.4 | 200 | 44.50 | CES-D | mild:10-16  moderate:17-24  moderate to severe: >24 | 2 |
| Perez-Lopez (2013)^[10]^ | Spain | cross-sectional | NR | NR | postmenopausal women | median=54  range:48-68 | 169 | 44.97 | CES-D | ≥10 | 3 |
| Tsiligianni (2014) ^[11]^ | Greek, Cyprus, Malta | cross-sectional | NR | random, multistage sampling | postmenopausal women | mean=74  SD=7 | 851 | 15.63 | GDS | >11 | 5 |
| Stanisławska (2014)^[12]^ | Poland | cross-sectional | NR | systematic sampling | postmenopausal women | mean=56  SD=6 | 171 | 32.16 | BDI | ≥20 | 4 |
| Singh (2014)^[13]^ | Iran | cross-sectional | 2011.10-2013.03 | simple random sampling | postmenopausal women | range: 40-54  mean=49.05  SD=3.85 | 252 | 32.14 | HAM-D | NR | 6 |
| Gangwisch (2015)^[14]^ | America | cross-sectional | 1994.09-1998.12 | NR | postmenopausal women | range:50-79 | 69954 | 6.64 | Burnam 8-item scale | ≥0.06 | 5 |
| Jung (2015) a^[15]^ | Korea | cross-sectional | 2010-2012 | multi-stage clustered probability sampling | postmenopausal women | mean=64.15  range:56-71 | 4869 | 5.67 | diagnosed by a physician | / | 8 |
| Jung (2015) b^[16]^ | Korea | cross-sectional | 2004-2012 | NR | postmenopausal women | range:35-74 | 60119 | 2.23 | CES-D | ≥21 | 7 |
| Afshari (2015)^[17]^ | Iran | cross-sectional | 2013-2014 | randomly sampling | postmenopausal women | range:40-65  mean=55. 27  SD=3.03 | 1280 | 59.14 | HAM-D | mild:8-13  moderate:14-18  severe: ≥19 | 5 |
| Slopień (2016)^[18]^ | Poland | cross-sectional | NR | NR | postmenopausal women | range:47-65  mean=54.6  SD=3.8 | 128 | 68.75 | HAM-D | NR | 1 |
| Szkup^[19]^ (2017) | Poland | cross-sectional | NR | NR | postmenopausal women | range:42-70  mean=56.26  SD=5.55  median=55 | 198 | 35.35 | BDI | mild:12-26  moderate:27-49  heavy:50-63 | 4 |
| Ho^[20]^ (2017) | China | cross-sectional | 2002-2004 | simple-random sampling | postmenopausal women | range: 50-64  mean=55.85  SD=3.24 | 379 | 11.87 | CES-D | ≥16 | 8 |
| Xiong^[21]^ (2017) | China | cross-sectional | 2013.11-2014.07 | convenience sampling | postmenopausal women | range: 42-76  mean=58.4  SD=5.0 | 437 | 26.77 | SDS | ≥53 | 9 |
| Heidari (2017)^[22]^ | Iran | cross-sectional | NR | convenience sampling | postmenopausal women | range:45-60 | 300 | 66.67 | DASS-21 | NR | 4 |
| Park (2018)^[23]^ | Korea | cross-sectional | 2013-2015 | stratified multistage cluster sampling | postmenopausal women | mean=64.7  SD=9.1 | 3860 | 4.61 | diagnosed by a physician | / | 6 |
| Dutta (2018)^[24]^ | India | cross-sectional | 2017.05-2017.07 | multi-stage sampling | postmenopausal women | mean=54.8 | 171 | 24.56 | HAM-D | mild:8-13  moderate:14-18  severe:19-22 | 5 |
| Park^[25]^ (2019) | Korea | cross-sectional | 2014 | stratified and multistage probability cluster sampling | postmenopausal women | mean=63.4 | 1372 | 10.13 | PHQ-9 | ≥10 | 6 |
| Wie (2019)^[26]^ | Korea | cross-sectional | 2010-2012 | stratified, multistage, probability sampling | postmenopausal women | mean=62.8 | 5131 | 25.06 | diagnosed by a physician | / | 5 |
| Liao^[27]^ (2019) | China | cross-sectional | 2013-2016 | NR | postmenopausal women | mean=58.8  SD=7.4 | 2051 | 11.31 | SDS | ≥48 | 8 |
| Nayak^[28]^ (2019) | India | cross-sectional | 2017.12-2018.01 | simple random sampling | postmenopausal women | mean=51.68  SD=4.254 | 290 | 22.41 | IDS-SR | mild:14-25  moderate:26-38  severe:39-48  very severe :49-84 | 5 |
| Ahlawat (2019)^[29]^ | India | cross-sectional | NR | systematic random sampling | postmenopausal women | range:41-60 | 580 | 41.55 | HAM-D | mild:8-13  moderate:14-18  severe:19-22  very severe depression: ≥23 | 3 |
| Li (2019)^[30]^ | China | cross-sectional | 2014-2015 | stratified randomly sampling | postmenopausal women | mean=69.2  SD=7.5 | 5537 | 12.01 | PHQ-9 | ≥5 | 8 |
| Ozdemir^[31]^ (2020) | Turkey | cross-sectional | 2018.03-09 | NR | postmenopausal women | mean=56.33  SD=7.34  range: 35-78 | 485 | 41.03 | BDI | ＞17 | 4 |
| Esmaeilzadeh (2020)^[32]^ | Iran | cross-sectional | 2016.09-2018.08 | cluster sampling | postmenopausal women | range:45-65  mean=54.3  SD=4.1 | 132 | 53.79 | BDI | mild:5‑7  moderate:8‑15  severe: ≥16 | 5 |
| Simbar^[33]^ (2020) | Iran | cross-sectional | NR | multi-stage sampling | postmenopausal women | mean=55.19  SD=4.03 | 307 | 55.05 | BDI | ＞10 | 4 |
| Wieder-Huszla (2020)^[34]^ | Poland | cross-sectional | NR | NR | postmenopausal women | range:45-75  mean=56.69  SD=6.0 | 102 | 22.55 | BDI | mild:12-26  moderate:27-49  severe:50-63 | 3 |
| Chae (2021)^[35]^ | Korea | cross-sectional | 2013-2015 | multi-stage stratified cluster sampling | postmenopausal women | mean=64.28  SD=0.3 | 4150 | 8.80 | diagnosed by a physician | / | 7 |
| Barghandan(2021)^[36]^ | Iran | cross-sectional | 2018.01-06 | cluster random sampling | postmenopausal women | mean=55.33  SD=4.48 | 245 | 89.39 | BDI | mild:14-19  moderate:20-28  severe:29-63 | 7 |
| Zhou (2021)^[37]^ | China | cross-sectional | 2011.06-2012.03 | multi-stage probability sampling | postmenopausal women | no depression: mean=62.36  SD=9.21  depression: mean=62.13 SD=9.21 | 2378 | 58.41 | CES-D | ≥10 | 7 |
| Wu (2021)^[38]^ | China | cross-sectional | 2017.07-2019.12 | NR | postmenopausal women | range:40-65  median=53 | 190 | 44.70 | CES-D | ≥16 | 5 |
| Chandankhede (2021)^[39]^ | India | cross-sectional | 2018.01-2019.12 | random sampling | postmenopausal women | range: 40-59 | 100 | 21.00 | SDS | ＞45 | 5 |
| Papazisis (2022)^[40]^ | Greece | cross-sectional | 2020.06-08 | NR | postmenopausal women | range:45-70  median=53 | 502 | 27.09 | BDI | ≥20 | 5 |
| Duzgun (2022)^[41]^ | Turkey | cross-sectional | 2020.02-06 | NR | postmenopausal women | range:51-72  mean=52.64  SD=6.245 | 242 | 26.03 | BDI | ≥17 | 5 |
| Alshogran (2022)^[42]^ | Jordan | cross-sectional | 2018.11-2019.03 | NR | postmenopausal women | range:44-77  mean=58.02  SD=5.8 | 450 | 14.50 | HADS | ≥11 | 6 |
| Hooper (2022)^[43]^ | America | cross-sectional | NR | snowball sampling | postmenopausal women | range: 60-94  mean=68.84  SD=6.53 | 227 | 23.79 | CES-D | ≥10 | 3 |
| Tong (2023)^[44]^ | China | cross-sectional | 2018 | multi-stage random cluster sampling | postmenopausal women | range:50-64  mean=58.022  SD=3.723 | 1465 | 26.83 | PHQ-9 | ≥5 | 8 |
| Wassertheil-Smoller (2004)^[45]^ | America | cohort | 1993.09-1998.12 | NR | postmenopausal women | range:50-79 | 93676 | 15.80 | CES-D (6 items) | ≥5 | 6 |
| Ryan (2009)^[46]^ | Australia | cohort | 1991-2003 | random sampling | postmenopausal women | mean=60.1  range:55.9-66.8 | 138 | 25.36 | CES-D | ≥10 | 8 |
| Colangelo (2012)^[47]^ | America | cohort | 2000-2005 | NR | postmenopausal women | range:45-84 | 1824 | 13.10 | CES-D | ≥16 | 9 |
| Perquier (2013)^[48]^ | France | cohort | 1990-2005 | NR | postmenopausal women | mean=63.8  SD=6.1 | 51088 | 11.63 | CES-D | ≥23 | 7 |
| Perquier (2014)^[49]^ | France | cohort | 1990-2005 | NR | postmenopausal women | mean=63.9  SD=6.2 | 41144 | 15.40 | CES-D | ≥23 | 6 |
| Persons (2016)^[50]^ | America | cohort | 1993-1998 | NR | postmenopausal women | range:50-79 | 20942 | 7.18 | CES-D (8-item) / DIS | ≥0.06 | 8 |

BDI, Beck Depressive Inventory; CES-D, Center for Epidemiologic Studies Depression Scale; GDS, Geriatric Depression Scale; HAM-D, Hamilton Depression scale; DASS-21, Depression Anxiety Stress Scale 21; SDS, Zung’s Self-rating Depression Scale; PHQ-9, 9-item Patient Health Questionnaire; DIS, Diagnostic Interview Schedule; ICD-10, Disease and Related Health Problems 10th revision; IDS-SR, Inventory of Depressive Symptomatology-Self Report scale; HADS, The hospital anxiety and depression scale; NR, Not Reported; SD, Standard Deviation

**References**

[1] BARRETT-CONNOR E, VON MÜHLEN D, LAUGHLIN G A, et al. Endogenous levels of dehydroepiandrosterone sulfate, but not other sex hormones, are associated with depressed mood in older women: the Rancho Bernardo Study [J]. J Am Geriatr Soc, 1999, 47(6): 685-91.

[2] KIM C K, MCGORRAY S P, BARTHOLOMEW B A, et al. Depressive symptoms and heart rate variability in postmenopausal women [J]. Arch Intern Med, 2005, 165(11): 1239-44.

[3] DEVECI S E, AÇIK Y, DAG D G, et al. The frequency of depression and menopause-related symptoms in postmenopausal women living in a province in Eastern Turkey, and the factors that affect depressive status [J]. Med Sci Monit, 2010, 16(4): Ph40-7.

[4] INA K, HAYASHI T, NOMURA H, et al. Depression, quality of life (QoL) and will to live of community-dwelling postmenopausal women in three Asian countries: Korea, China and Japan [J]. Arch Gerontol Geriatr, 2011, 53(1): 8-12.

[5] UNSAL A, TOZUN M, AYRANCI U. Prevalence of depression among postmenopausal women and related characteristics [J]. Climacteric, 2011, 14(2): 244-51.

[6] HUMENIUK E, BOJAR I, OWOC A, et al. Psychosocial conditioning of depressive disorders in post-menopausal women [J]. Ann Agric Environ Med, 2011, 18(2): 441-5.

[7] SEIB C, ANDERSON D, LEE K, et al. Predictors of mental health in post-menopausal women: results from the Australian healthy aging of women study [J]. Maturitas, 2013, 76(4): 377-83.

[8] GROCHANS E, GRZYWACZ A, JURCZAK A, et al. The 5HTT and MAO-A polymorphisms associate with depressive mood and climacteric symptoms in postmenopausal women [J]. Prog Neuropsychopharmacol Biol Psychiatry, 2013, 45: 125-30.

[9] TAMARIA A, BHARTI R, SHARMA M, et al. Risk assessment for psychological disorders in postmenopausal women [J]. J Clin Diagn Res, 2013, 7(12): 2885-8.

[10] PEREZ-LOPEZ F R, PEREZ-RONCERO G, FERNANDEZ-INARREA J, et al. Resilience, depressed mood, and menopausal symptoms in postmenopausal women [J]. Menopause-the Journal of the North American Menopause Society, 2014, 21(2): 159-64.

[11] TSILIGIANNI I G, TYROVOLAS S, BOUNTZIOUKA V, et al. Depressive symptoms in postmenopausal women: results from the MEDIS Study [J]. Women Health, 2014, 54(5): 389-401.

[12] STANISŁAWSKA M, SZKUP-JABŁOŃSKA M, JURCZAK A, et al. The severity of depressive symptoms vs. serum Mg and Zn levels in postmenopausal women [J]. Biol Trace Elem Res, 2014, 157(1): 30-5.

[13] SINGH A, PRADHAN S K. Menopausal symptoms of postmenopausal women in a rural community of Delhi, India: A cross-sectional study [J]. J Midlife Health, 2014, 5(2): 62-7.

[14] GANGWISCH J E, HALE L, GARCIA L, et al. High glycemic index diet as a risk factor for depression: analyses from the Women's Health Initiative [J]. Am J Clin Nutr, 2015, 102(2): 454-63.

[15] JUNG S J, SHIN A, KANG D. Hormone-related factors and post-menopausal onset depression: results from KNHANES (2010-2012) [J]. J Affect Disord, 2015, 175: 176-83.

[16] JUNG S J, SHIN A, KANG D. Menarche age, menopause age and other reproductive factors in association with post-menopausal onset depression: Results from Health Examinees Study (HEXA) [J]. J Affect Disord, 2015, 187: 127-35.

[17] AFSHARI P, MANOCHEHRI S, TADAYON M, et al. Prevalence of depression in postmenopausal women [J]. Jundishapur Journal of Chronic Disease Care, 2015, 4(3).

[18] SLOPIEŃ R, SLOPIEŃ A, PAWLAK M, et al. Depressive symptoms' pattern in postmenopausal women [J]. Clin Exp Obstet Gynecol, 2016, 43(4): 544-5.

[19] SZKUP M, JURCZAK A, BRODOWSKA A, et al. Analysis of Relations Between the Level of Mg, Zn, Ca, Cu, and Fe and Depressiveness in Postmenopausal Women [J]. Biol Trace Elem Res, 2017, 176(1): 56-63.

[20] HO S C, LIANG Z, YU R H, et al. Association of life events and depressive symptoms among early postmenopausal Chinese women in Hong Kong [J]. Menopause, 2017, 24(2): 180-6.

[21] XIONG Q, HU X, XU Y, et al. Association of visceral fat area with the presence of depressive symptoms in Chinese postmenopausal women with normal glucose tolerance [J]. Menopause, 2017, 24(11): 1289-94.

[22] HEIDARI M, GHODUSI M, RAFIEI H. Sexual Self-concept and Its Relationship to Depression, Stress and Anxiety in Postmenopausal Women [J]. J Menopausal Med, 2017, 23(1): 42-8.

[23] PARK H, KIM K. Depression and Its Association with Health-Related Quality of Life in Postmenopausal Women in Korea [J]. Int J Environ Res Public Health, 2018, 15(11).

[24] DUTTA R, RAJENDRAN P, RAMYA S, et al. Prevalence of depression among the post-menopausal women in the field practice area of Saveetha medical college and hospital, Thirumazhisai, Tamil Nadu [J]. Indian Journal of Public Health Research and Development, 2018, 9(11): 175-9.

[25] PARK S, CHOI N-K. Breastfeeding reduces risk of depression later in life in the postmenopausal period: A Korean population-based study [J]. Journal of Affective Disorders, 2019, 248: 13-7.

[26] WIE J H, NAM S K, KO H S, et al. The association between abortion experience and postmenopausal suicidal ideation and mental health: Results from the 5th Korean National Health and Nutrition Examination Survey (KNHANES V) [J]. Taiwan J Obstet Gynecol, 2019, 58(1): 153-8.

[27] LIAO K, GU Y, LIU M, et al. Association of dietary patterns with depressive symptoms in Chinese postmenopausal women [J]. Br J Nutr, 2019, 122(10): 1168-74.

[28] NAYAK S, BINIL V, CHRISTABEL S. Depressive symptoms and bio-psychosocial problems among postmenopausal women of Udupi district, Karnataka, India [J]. Journal of Clinical and Diagnostic Research, 2019, 13(1): VC01-VC4.

[29] AHLAWAT P, SINGH M M, GARG S, et al. Prevalence of Depression and its Association with Sociodemographic Factors in Postmenopausal Women in an Urban Resettlement Colony of Delhi [J]. J Midlife Health, 2019, 10(1): 33-6.

[30] LI F, HE F, SUN Q, et al. Reproductive history and risk of depressive symptoms in postmenopausal women: A cross-sectional study in eastern China [J]. J Affect Disord, 2019, 246: 174-81.

[31] OZDEMIR K, SAHIN S, GULER D S, et al. Depression, anxiety, and fear of death in postmenopausal women [J]. Menopause, 2020, 27(9): 1030-6.

[32] ESMAEILZADEH S, AGAJANI DELAVAR M, NOURI H, et al. Examination of associations between personality traits, and polymorphisms of MAO-A and 5-HTT with the severity of menopausal symptoms and depression levels [J]. Biomed Rep, 2020, 12(5): 259-66.

[33] SIMBAR M, NAZARPOUR S, ALAVI MAJD H, et al. Is body image a predictor of women's depression and anxiety in postmenopausal women? [J]. BMC Psychiatry, 2020, 20(1): 202.

[34] WIEDER-HUSZLA S, ZABIELSKA P, KOTWAS A, et al. THE SEVERITY OF DEPRESSIVE AND ANXIETY SYMPTOMS IN POSTMENOPAUSAL WOMEN DEPENDING ON THEIR MAGNESIUM, ZINC, SELENIUM AND COPPER LEVELS [J]. Journal of Elementology, 2020, 25(4): 1305-17.

[35] CHAE M, PARK K. Association between dietary omega-3 fatty acid intake and depression in postmenopausal women [J]. Nutr Res Pract, 2021, 15(4): 468-78.

[36] BARGHANDAN N, DOLATKHAH N, ESLAMIAN F, et al. Association of depression, anxiety and menopausal-related symptoms with demographic, anthropometric and body composition indices in healthy postmenopausal women [J]. BMC Womens Health, 2021, 21(1): 192.

[37] ZHOU Z, YU Y, ZHOU R, et al. Associations between sleep duration, midday napping, depression, and falls among postmenopausal women in China: a population-based nationwide study [J]. Menopause, 2021, 28(5): 554-63.

[38] WU Y T, HUANG W Y, KOR C T, et al. Relationships between depression and anxiety symptoms and adipocyte-derived proteins in postmenopausal women [J]. PLoS One, 2021, 16(3): e0248314.

[39] CHANDANKHEDE M, GUPTA M, PAKHMODE S. Assessment of Psychological Status and Oxidative Stress in Postmenopausal Women: A Cross-Sectional Study [J]. J Menopausal Med, 2021, 27(3): 155-61.

[40] PAPAZISIS G, TSAKIRIDIS I, AINATZOGLOU A, et al. Prevalence of post-menopausal depression and associated factors: A web-based cross-sectional study in Greece [J]. Maturitas, 2022, 156: 12-7.

[41] DUZGUN A A, KOK G, SAHIN S, et al. Assessment of depression and sexual quality of life in postmenopausal women [J]. Perspect Psychiatr Care, 2022, 58(4): 2029-36.

[42] ALSHOGRAN O Y, MAHMOUD F M Z, ALKHATATBEH M J. Predictors of age at menopause and psychiatric symptoms among postmenopausal females in Jordan [J]. J Psychosom Obstet Gynaecol, 2022, 43(4): 385-92.

[43] HOOPER S C, MARSHALL V B, BECKER C B, et al. Mental health and quality of life in postmenopausal women as a function of retrospective menopause symptom severity [J]. Menopause, 2022, 29(6): 707-13.

[44] TONG C, MENG Y, LI T, et al. High levels of physical activity are associated with a reduced likelihood of depressive symptoms in postmenopausal women [J]. Women Health, 2023: 1-11.

[45] WASSERTHEIL-SMOLLER S, SHUMAKER S, OCKENE J, et al. Depression and cardiovascular sequelae in postmenopausal women. The Women's Health Initiative (WHI) [J]. Arch Intern Med, 2004, 164(3): 289-98.

[46] RYAN J, BURGER H G, SZOEKE C, et al. A prospective study of the association between endogenous hormones and depressive symptoms in postmenopausal women [J]. Menopause-the Journal of the North American Menopause Society, 2009, 16(3): 509-17.

[47] COLANGELO L A, CRAFT L L, OUYANG P, et al. Association of sex hormones and sex hormone-binding globulin with depressive symptoms in postmenopausal women: the Multiethnic Study of Atherosclerosis [J]. Menopause, 2012, 19(8): 877-85.

[48] PERQUIER F, RYAN J, ANCELIN M-L, et al. Lifetime endogenous reproductive factors and severe depressive symptoms in postmenopausal women: findings from the E3N cohort [J]. Menopause-the Journal of the North American Menopause Society, 2013, 20(11): 1154-63.

[49] PERQUIER F, LASFARGUES A, MESRINE S, et al. Body-size throughout life and risk of depression in postmenopausal women: findings from the E3N cohort [J]. Obesity (Silver Spring), 2014, 22(8): 1926-34.

[50] PERSONS J E, ROBINSON J G, CORYELL W H, et al. Longitudinal study of low serum LDL cholesterol and depressive symptom onset in postmenopause [J]. J Clin Psychiatry, 2016, 77(2): 212-20.
