# Supplementary material for: Prevalence and associated factors of depression in postmenopausal women: a systematic review and meta-analysis
Source: BMC Psychiatry. 2024 Jun 10;24:431. doi: 10.1186/s12888-024-05875-0 (PMC11165857; doi:10.1186/s12888-024-05875-0)
Supplement: Supplementary file 4 — Supplementary Material 4: Quality assessment of included studies [file 12888_2024_5875_MOESM4_ESM.docx]

**Additional file 4:**

**Table 2 (A) Quality assessment of including cross-sectional studies**

| author | total quality score | 1) Define the source of information (survey, record review) | 2) List inclusion and exclusion criteria for exposed and unexposed subjects (cases and controls) or refer to previous publications | 3) Indicate time period used for identifying patients | 4) Indicate whether or not subjects were consecutive if not population-based | 5) Indicate if evaluators of subjective components of study were masked to other aspects of the status of the participants | 6) Describe any assessments undertaken for quality assurance purposes | 7) Explain any patient exclusions from analysis | 8) Describe how confounding was assessed and/or controlled. | 9) If applicable, explain how missing data were handled in the analysis | 10) Summarize patient response rates and completeness of data collection | 11) Clarify what follow-up, if any, was expected and the percentage of patients for which incomplete data or follow-up was obtained |
| --- | --- | --- | --- | --- | --- | --- | --- | --- | --- | --- | --- | --- |
| Szkup^[1]^ | 4 | 1 | 1 | 0 | 0 | 1 | 1 | 0 | 0 | 0 | 0 | 0 |
| Duzgun^[2]^ | 5 | 1 | 1 | 1 | 1 | 1 | 0 | 0 | 0 | 0 | 0 | 0 |
| Wie^[3]^ | 5 | 1 | 0 | 1 | 1 | 1 | 0 | 0 | 1 | 0 | 0 | 0 |
| Chae^[4]^ | 7 | 1 | 1 | 1 | 1 | 1 | 1 | 0 | 1 | 0 | 0 | 0 |
| Barghandan^[5]^ | 7 | 1 | 1 | 1 | 1 | 1 | 1 | 0 | 0 | 0 | 1 | 0 |
| Liao^[6]^ | 8 | 1 | 1 | 1 | 1 | 1 | 1 | 0 | 1 | 0 | 1 | 0 |
| Ho^[7]^ | 8 | 1 | 1 | 1 | 1 | 1 | 0 | 0 | 1 | 0 | 1 | 1 |
| Xiong^[8]^ | 9 | 1 | 1 | 1 | 1 | 1 | 1 | 1 | 1 | 0 | 1 | 0 |
| Park^[9]^ | 6 | 1 | 0 | 1 | 1 | 1 | 0 | 0 | 1 | 0 | 1 | 0 |
| Ozdemir^[10]^ | 4 | 1 | 0 | 1 | 1 | 1 | 0 | 0 | 0 | 0 | 0 | 0 |
| Ina^[11]^ | 2 | 1 | 0 | 0 | 0 | 0 | 0 | 0 | 0 | 0 | 1 | 0 |
| Nayak^[12]^ | 5 | 1 | 1 | 1 | 1 | 1 | 0 | 0 | 0 | 0 | 0 | 0 |
| Kim^[13]^ | 4 | 1 | 1 | 1 | 0 | 0 | 0 | 0 | 0 | 0 | 1 | 0 |
| Tsiligianni^[14]^ | 5 | 1 | 1 | 0 | 0 | 1 | 0 | 0 | 1 | 0 | 1 | 0 |
| Slopień^[15]^ | 1 | 1 | 0 | 0 | 0 | 0 | 0 | 0 | 0 | 0 | 0 | 0 |
| Barrett-Connor^[16]^ | 6 | 1 | 0 | 1 | 1 | 1 | 0 | 0 | 1 | 0 | 1 | 0 |
| Esmaeilzadeh^[17]^ | 5 | 1 | 1 | 1 | 1 | 1 | 0 | 0 | 0 | 0 | 0 | 0 |
| Deveci^[18]^ | 3 | 1 | 0 | 0 | 0 | 1 | 0 | 0 | 0 | 0 | 1 | 0 |
| Gangwisch^[19]^ | 5 | 1 | 1 | 1 | 1 | 0 | 0 | 0 | 1 | 0 | 0 | 0 |
| Tong^[20]^ | 8 | 1 | 1 | 1 | 1 | 1 | 0 | 0 | 1 | 1 | 1 | 0 |
| Jung^[21]^ | 8 | 1 | 1 | 1 | 1 | 1 | 0 | 0 | 1 | 1 | 1 | 0 |
| Perez-Lopez ^[22]^ | 3 | 1 | 1 | 0 | 0 | 0 | 0 | 0 | 0 | 0 | 1 | 0 |
| Simbar^[23]^ | 4 | 1 | 1 | 0 | 0 | 0 | 0 | 0 | 1 | 0 | 1 | 0 |
| Jung^[24]^ | 7 | 1 | 1 | 1 | 1 | 1 | 0 | 0 | 1 | 0 | 1 | 0 |
| Alshogran^[25]^ | 6 | 1 | 1 | 1 | 1 | 1 | 0 | 0 | 0 | 0 | 1 | 0 |
| Seib^[26]^ | 8 | 1 | 1 | 1 | 1 | 1 | 0 | 0 | 0 | 1 | 1 | 1 |
| Unsal^[27]^ | 6 | 1 | 1 | 1 | 1 | 1 | 0 | 1 | 0 | 0 | 0 | 0 |
| Dutta^[28]^ | 5 | 1 | 1 | 1 | 1 | 1 | 0 | 0 | 0 | 0 | 0 | 0 |
| Zhou^[29]^ | 7 | 1 | 0 | 1 | 1 | 1 | 0 | 0 | 1 | 1 | 1 | 0 |
| Grochans^[30]^ | 3 | 1 | 1 | 0 | 0 | 1 | 0 | 0 | 0 | 0 | 0 | 0 |
| Park^[31]^ | 6 | 1 | 0 | 1 | 1 | 1 | 0 | 0 | 1 | 0 | 1 | 0 |
| Papazisis^[32]^ | 5 | 1 | 0 | 1 | 1 | 1 | 0 | 0 | 0 | 0 | 1 | 0 |
| Heidari^[33]^ | 4 | 1 | 1 | 0 | 1 | 1 | 0 | 0 | 0 | 0 | 0 | 0 |
| Wieder-Huszla^[34]^ | 3 | 1 | 1 | 0 | 0 | 1 | 0 | 0 | 0 | 0 | 0 | 0 |
| Stanisławska^[35]^ | 4 | 1 | 1 | 0 | 0 | 1 | 1 | 0 | 0 | 0 | 0 | 0 |
| Wu^[36]^ | 5 | 1 | 1 | 1 | 1 | 0 | 0 | 0 | 1 | 0 | 0 | 0 |
| Ahlawat^[37]^ | 3 | 1 | 1 | 0 | 0 | 1 | 0 | 0 | 0 | 0 | 0 | 0 |
| Humeniuk^[38]^ | 7 | 1 | 1 | 1 | 1 | 1 | 0 | 1 | 0 | 0 | 1 | 0 |
| Li^[39]^ | 8 | 1 | 1 | 1 | 1 | 1 | 0 | 0 | 1 | 1 | 1 | 0 |
| Singh^[40]^ | 6 | 1 | 1 | 1 | 1 | 1 | 0 | 0 | 0 | 0 | 1 | 0 |
| Hooper^[41]^ | 3 | 1 | 0 | 0 | 0 | 1 | 0 | 0 | 1 | 0 | 0 | 0 |
| Tamaria^[42]^ | 2 | 1 | 1 | 0 | 0 | 0 | 0 | 0 | 0 | 0 | 0 | 0 |
| Chandankhede^[43]^ | 5 | 1 | 1 | 1 | 1 | 1 | 0 | 0 | 0 | 0 | 0 | 0 |
| Afshari^[44]^ | 5 | 1 | 1 | 1 | 1 | 1 | 0 | 0 | 0 | 0 | 0 | 0 |

**Table 2 (B) Quality assessment of including longitudinal studies**

| author | total quality score | 1) Representativeness of the exposed cohort | 2) Selection of the non exposed cohort | 3) Ascertainment of exposure | 4) Demonstration that outcome of interest was no present as start of study | 5) Comparability of cohort on the basis of the design or analysis | 6) Assessment of outcome | 7) Was follow-up long enough for outcomes to occur | 8)Adequacy of follow up of cohorts |
| --- | --- | --- | --- | --- | --- | --- | --- | --- | --- |
| Colangelo^[45]^ | 9 | 1 | 1 | 1 | 1 | 2 | 1 | 1 | 1 |
| Perquier^[46]^ | 6 | 0 | 1 | 1 | 1 | 2 | 0 | 1 | 0 |
| Wassertheil-Smoller^[47]^ | 5 | 0 | 1 | 1 | 0 | 2 | 0 | 1 | 0 |
| Persons^[48]^ | 8 | 0 | 1 | 1 | 1 | 2 | 1 | 1 | 1 |
| Ryan^[49]^ | 8 | 1 | 1 | 1 | 0 | 2 | 1 | 1 | 1 |
| Perquier^[50]^ | 7 | 0 | 1 | 2 | 0 | 2 | 1 | 1 | 1 |

**References**

[1] SZKUP M, JURCZAK A, BRODOWSKA A, et al. Analysis of Relations Between the Level of Mg, Zn, Ca, Cu, and Fe and Depressiveness in Postmenopausal Women [J]. Biol Trace Elem Res, 2017, 176(1): 56-63.

[2] DUZGUN A A, KOK G, SAHIN S, et al. Assessment of depression and sexual quality of life in postmenopausal women [J]. Perspect Psychiatr Care, 2022, 58(4): 2029-36.

[3] WIE J H, NAM S K, KO H S, et al. The association between abortion experience and postmenopausal suicidal ideation and mental health: Results from the 5th Korean National Health and Nutrition Examination Survey (KNHANES V) [J]. Taiwan J Obstet Gynecol, 2019, 58(1): 153-8.

[4] CHAE M, PARK K. Association between dietary omega-3 fatty acid intake and depression in postmenopausal women [J]. Nutr Res Pract, 2021, 15(4): 468-78.

[5] BARGHANDAN N, DOLATKHAH N, ESLAMIAN F, et al. Association of depression, anxiety and menopausal-related symptoms with demographic, anthropometric and body composition indices in healthy postmenopausal women [J]. BMC Womens Health, 2021, 21(1): 192.

[6] LIAO K, GU Y, LIU M, et al. Association of dietary patterns with depressive symptoms in Chinese postmenopausal women [J]. Br J Nutr, 2019, 122(10): 1168-74.

[7] HO S C, LIANG Z, YU R H, et al. Association of life events and depressive symptoms among early postmenopausal Chinese women in Hong Kong [J]. Menopause, 2017, 24(2): 180-6.

[8] XIONG Q, HU X, XU Y, et al. Association of visceral fat area with the presence of depressive symptoms in Chinese postmenopausal women with normal glucose tolerance [J]. Menopause, 2017, 24(11): 1289-94.

[9] PARK S, CHOI N-K. Breastfeeding reduces risk of depression later in life in the postmenopausal period: A Korean population-based study [J]. Journal of Affective Disorders, 2019, 248: 13-7.

[10] OZDEMIR K, SAHIN S, GULER D S, et al. Depression, anxiety, and fear of death in postmenopausal women [J]. Menopause, 2020, 27(9): 1030-6.

[11] INA K, HAYASHI T, NOMURA H, et al. Depression, quality of life (QoL) and will to live of community-dwelling postmenopausal women in three Asian countries: Korea, China and Japan [J]. Arch Gerontol Geriatr, 2011, 53(1): 8-12.

[12] NAYAK S, BINIL V, CHRISTABEL S. Depressive symptoms and bio-psychosocial problems among postmenopausal women of Udupi district, Karnataka, India [J]. Journal of Clinical and Diagnostic Research, 2019, 13(1): VC01-VC4.

[13] KIM C K, MCGORRAY S P, BARTHOLOMEW B A, et al. Depressive symptoms and heart rate variability in postmenopausal women [J]. Arch Intern Med, 2005, 165(11): 1239-44.

[14] TSILIGIANNI I G, TYROVOLAS S, BOUNTZIOUKA V, et al. Depressive symptoms in postmenopausal women: results from the MEDIS Study [J]. Women Health, 2014, 54(5): 389-401.

[15] SLOPIEŃ R, SLOPIEŃ A, PAWLAK M, et al. Depressive symptoms' pattern in postmenopausal women [J]. Clin Exp Obstet Gynecol, 2016, 43(4): 544-5.

[16] BARRETT-CONNOR E, VON MÜHLEN D, LAUGHLIN G A, et al. Endogenous levels of dehydroepiandrosterone sulfate, but not other sex hormones, are associated with depressed mood in older women: the Rancho Bernardo Study [J]. J Am Geriatr Soc, 1999, 47(6): 685-91.

[17] ESMAEILZADEH S, AGAJANI DELAVAR M, NOURI H, et al. Examination of associations between personality traits, and polymorphisms of MAO-A and 5-HTT with the severity of menopausal symptoms and depression levels [J]. Biomed Rep, 2020, 12(5): 259-66.

[18] DEVECI S E, AÇIK Y, DAG D G, et al. The frequency of depression and menopause-related symptoms in postmenopausal women living in a province in Eastern Turkey, and the factors that affect depressive status [J]. Med Sci Monit, 2010, 16(4): Ph40-7.

[19] GANGWISCH J E, HALE L, GARCIA L, et al. High glycemic index diet as a risk factor for depression: analyses from the Women's Health Initiative [J]. Am J Clin Nutr, 2015, 102(2): 454-63.

[20] TONG C, MENG Y, LI T, et al. High levels of physical activity are associated with a reduced likelihood of depressive symptoms in postmenopausal women [J]. Women Health, 2023: 1-11.

[21] JUNG S J, SHIN A, KANG D. Hormone-related factors and post-menopausal onset depression: results from KNHANES (2010-2012) [J]. J Affect Disord, 2015, 175: 176-83.

[22] PEREZ-LOPEZ F R, PEREZ-RONCERO G, FERNANDEZ-INARREA J, et al. Resilience, depressed mood, and menopausal symptoms in postmenopausal women [J]. Menopause-the Journal of the North American Menopause Society, 2014, 21(2): 159-64.

[23] SIMBAR M, NAZARPOUR S, ALAVI MAJD H, et al. Is body image a predictor of women's depression and anxiety in postmenopausal women? [J]. BMC Psychiatry, 2020, 20(1): 202.

[24] JUNG S J, SHIN A, KANG D. Menarche age, menopause age and other reproductive factors in association with post-menopausal onset depression: Results from Health Examinees Study (HEXA) [J]. J Affect Disord, 2015, 187: 127-35.

[25] ALSHOGRAN O Y, MAHMOUD F M Z, ALKHATATBEH M J. Predictors of age at menopause and psychiatric symptoms among postmenopausal females in Jordan [J]. J Psychosom Obstet Gynaecol, 2022, 43(4): 385-92.

[26] SEIB C, ANDERSON D, LEE K, et al. Predictors of mental health in post-menopausal women: results from the Australian healthy aging of women study [J]. Maturitas, 2013, 76(4): 377-83.

[27] UNSAL A, TOZUN M, AYRANCI U. Prevalence of depression among postmenopausal women and related characteristics [J]. Climacteric, 2011, 14(2): 244-51.

[28] DUTTA R, RAJENDRAN P, RAMYA S, et al. Prevalence of depression among the post-menopausal women in the field practice area of Saveetha medical college and hospital, Thirumazhisai, Tamil Nadu [J]. Indian Journal of Public Health Research and Development, 2018, 9(11): 175-9.

[29] ZHOU Z, YU Y, ZHOU R, et al. Associations between sleep duration, midday napping, depression, and falls among postmenopausal women in China: a population-based nationwide study [J]. Menopause, 2021, 28(5): 554-63.

[30] GROCHANS E, GRZYWACZ A, JURCZAK A, et al. The 5HTT and MAO-A polymorphisms associate with depressive mood and climacteric symptoms in postmenopausal women [J]. Prog Neuropsychopharmacol Biol Psychiatry, 2013, 45: 125-30.

[31] PARK H, KIM K. Depression and Its Association with Health-Related Quality of Life in Postmenopausal Women in Korea [J]. Int J Environ Res Public Health, 2018, 15(11).

[32] PAPAZISIS G, TSAKIRIDIS I, AINATZOGLOU A, et al. Prevalence of post-menopausal depression and associated factors: A web-based cross-sectional study in Greece [J]. Maturitas, 2022, 156: 12-7.

[33] HEIDARI M, GHODUSI M, RAFIEI H. Sexual Self-concept and Its Relationship to Depression, Stress and Anxiety in Postmenopausal Women [J]. J Menopausal Med, 2017, 23(1): 42-8.

[34] WIEDER-HUSZLA S, ZABIELSKA P, KOTWAS A, et al. THE SEVERITY OF DEPRESSIVE AND ANXIETY SYMPTOMS IN POSTMENOPAUSAL WOMEN DEPENDING ON THEIR MAGNESIUM, ZINC, SELENIUM AND COPPER LEVELS [J]. Journal of Elementology, 2020, 25(4): 1305-17.

[35] STANISŁAWSKA M, SZKUP-JABŁOŃSKA M, JURCZAK A, et al. The severity of depressive symptoms vs. serum Mg and Zn levels in postmenopausal women [J]. Biol Trace Elem Res, 2014, 157(1): 30-5.

[36] WU Y T, HUANG W Y, KOR C T, et al. Relationships between depression and anxiety symptoms and adipocyte-derived proteins in postmenopausal women [J]. PLoS One, 2021, 16(3): e0248314.

[37] AHLAWAT P, SINGH M M, GARG S, et al. Prevalence of Depression and its Association with Sociodemographic Factors in Postmenopausal Women in an Urban Resettlement Colony of Delhi [J]. J Midlife Health, 2019, 10(1): 33-6.

[38] HUMENIUK E, BOJAR I, OWOC A, et al. Psychosocial conditioning of depressive disorders in post-menopausal women [J]. Ann Agric Environ Med, 2011, 18(2): 441-5.

[39] LI F, HE F, SUN Q, et al. Reproductive history and risk of depressive symptoms in postmenopausal women: A cross-sectional study in eastern China [J]. J Affect Disord, 2019, 246: 174-81.

[40] SINGH A, PRADHAN S K. Menopausal symptoms of postmenopausal women in a rural community of Delhi, India: A cross-sectional study [J]. J Midlife Health, 2014, 5(2): 62-7.

[41] HOOPER S C, MARSHALL V B, BECKER C B, et al. Mental health and quality of life in postmenopausal women as a function of retrospective menopause symptom severity [J]. Menopause, 2022, 29(6): 707-13.

[42] TAMARIA A, BHARTI R, SHARMA M, et al. Risk assessment for psychological disorders in postmenopausal women [J]. J Clin Diagn Res, 2013, 7(12): 2885-8.

[43] CHANDANKHEDE M, GUPTA M, PAKHMODE S. Assessment of Psychological Status and Oxidative Stress in Postmenopausal Women: A Cross-Sectional Study [J]. J Menopausal Med, 2021, 27(3): 155-61.

[44] AFSHARI P, MANOCHEHRI S, TADAYON M, et al. Prevalence of depression in postmenopausal women [J]. Jundishapur Journal of Chronic Disease Care, 2015, 4(3).

[45] COLANGELO L A, CRAFT L L, OUYANG P, et al. Association of sex hormones and sex hormone-binding globulin with depressive symptoms in postmenopausal women: the Multiethnic Study of Atherosclerosis [J]. Menopause, 2012, 19(8): 877-85.

[46] PERQUIER F, LASFARGUES A, MESRINE S, et al. Body-size throughout life and risk of depression in postmenopausal women: findings from the E3N cohort [J]. Obesity (Silver Spring), 2014, 22(8): 1926-34.

[47] WASSERTHEIL-SMOLLER S, SHUMAKER S, OCKENE J, et al. Depression and cardiovascular sequelae in postmenopausal women. The Women's Health Initiative (WHI) [J]. Arch Intern Med, 2004, 164(3): 289-98.

[48] PERSONS J E, ROBINSON J G, CORYELL W H, et al. Longitudinal study of low serum LDL cholesterol and depressive symptom onset in postmenopause [J]. J Clin Psychiatry, 2016, 77(2): 212-20.

[49] RYAN J, BURGER H G, SZOEKE C, et al. A prospective study of the association between endogenous hormones and depressive symptoms in postmenopausal women [J]. Menopause-the Journal of the North American Menopause Society, 2009, 16(3): 509-17.

[50] PERQUIER F, RYAN J, ANCELIN M-L, et al. Lifetime endogenous reproductive factors and severe depressive symptoms in postmenopausal women: findings from the E3N cohort [J]. Menopause-the Journal of the North American Menopause Society, 2013, 20(11): 1154-63.
