# Supplementary material for: Prevalence and associated factors of depression in postmenopausal women: a systematic review and meta-analysis
Source: BMC Psychiatry. 2024 Jun 10;24:431. doi: 10.1186/s12888-024-05875-0 (PMC11165857; doi:10.1186/s12888-024-05875-0)
Supplement: Supplementary file 5 — Supplementary Material 5: Included studies of factors associated with depression symptoms [file 12888_2024_5875_MOESM5_ESM.docx]

**Additional file 5:**

**Table S3. Included studies of factors associated with depression symptoms.**

| **Associated factors** | **Included studies** |
| --- | --- |
| **Demographic characteristics** |  |
| age | Barghandan^[1]^, Papazisis^[2]^ |
| marital status | Ozdemir^[3]^, Deveci^[4]^, Unsal^[5]^ |
| number of children | Ozdemir^[3]^, Unsal^[5]^ |
| education | Unsal^[5]^ |
| working status | Papazisis^[2]^ |
| **Lifestyle factors** |  |
| weight | Perquier^[6]^, Wassertheil-Smoller^[7]^ |
| dietary pattern | Chae^[8]^, Liao^[9]^ , Gangwisch^[10]^ |
| physical activity | Barghandan^[1]^, Tong^[11]^, Wassertheil-Smoller^[7]^ |
| alcohol consumption | Ozdemir^[3]^ |
| smoking status | Papazisis^[2]^, Wassertheil-Smoller^[7]^ |
| **Medical and health factors** |  |
| history of mental illness | Ozdemir^[3]^, Unsal^[5]^, Papazisis^[2]^ |
| experience of violence | Deveci^[4]^ |
| life events | Ho^[12]^ |
| chronic disease | Ozdemir^[3]^, Deveci^[4]^ |
| disability | Ozdemir^[3]^ |
| cardiovascular disease events | Wassertheil-Smoller^[7]^ |
| serum low-density lipoprotein cholesterol | Persons^[13]^ |
| **Reproductive health factors** |  |
| menstrual cycle | Unsal^[5]^, Perquier^[14]^ |
| menstrual cycle length | Li^[15]^, Perquier^[14]^ |
| reproductive period | Jung^[16]^, Jung^[17]^, Li^[15]^ |
| full-term pregnancies | Jung^[16]^, Li^[15]^, Perquier^[14]^ |
| experienced pregnancy | Jung^[17]^, Li^[15]^ |
| abortion numbers | Jung^[16]^, Jung^[17]^ , Li^[15]^ |
| number of induced abortions | Wie^[18]^, Jung^[17]^ |
| number of breastfed infants | Park^[19]^, Jung^[17]^ |
| periods of breastfed infants | Park^[19]^ |
| contraceptive surgery | Li^[15]^ |
| menopause age | Tsiligianni^[20]^, Jung^[16]^, Jung^[17]^, Unsal^[5]^ |
| type of menopause | Jung^[17]^, Perquier^[14]^ |
| oral contraceptive usage | Jung^[16]^, Jung^[17]^ |
| hormone replacement therapy | Jung^[16]^, Jung^[17]^, Wassertheil-Smoller^[7]^ |
| sex hormones | Colangelo^[21]^, Ryan^[22]^ |
| menopausal symptoms | Tong^[11]^, Perquier^[14]^ |

**References**

[1] BARGHANDAN N, DOLATKHAH N, ESLAMIAN F, et al. Association of depression, anxiety and menopausal-related symptoms with demographic, anthropometric and body composition indices in healthy postmenopausal women [J]. BMC Womens Health, 2021, 21(1): 192.

[2] PAPAZISIS G, TSAKIRIDIS I, AINATZOGLOU A, et al. Prevalence of post-menopausal depression and associated factors: A web-based cross-sectional study in Greece [J]. Maturitas, 2022, 156: 12-7.

[3] OZDEMIR K, SAHIN S, GULER D S, et al. Depression, anxiety, and fear of death in postmenopausal women [J]. Menopause, 2020, 27(9): 1030-6.

[4] DEVECI S E, AÇIK Y, DAG D G, et al. The frequency of depression and menopause-related symptoms in postmenopausal women living in a province in Eastern Turkey, and the factors that affect depressive status [J]. Med Sci Monit, 2010, 16(4): Ph40-7.

[5] UNSAL A, TOZUN M, AYRANCI U. Prevalence of depression among postmenopausal women and related characteristics [J]. Climacteric, 2011, 14(2): 244-51.

[6] PERQUIER F, LASFARGUES A, MESRINE S, et al. Body-size throughout life and risk of depression in postmenopausal women: findings from the E3N cohort [J]. Obesity (Silver Spring), 2014, 22(8): 1926-34.

[7] WASSERTHEIL-SMOLLER S, SHUMAKER S, OCKENE J, et al. Depression and cardiovascular sequelae in postmenopausal women. The Women's Health Initiative (WHI) [J]. Arch Intern Med, 2004, 164(3): 289-98.

[8] CHAE M, PARK K. Association between dietary omega-3 fatty acid intake and depression in postmenopausal women [J]. Nutr Res Pract, 2021, 15(4): 468-78.

[9] LIAO K, GU Y, LIU M, et al. Association of dietary patterns with depressive symptoms in Chinese postmenopausal women [J]. Br J Nutr, 2019, 122(10): 1168-74.

[10] GANGWISCH J E, HALE L, GARCIA L, et al. High glycemic index diet as a risk factor for depression: analyses from the Women's Health Initiative [J]. Am J Clin Nutr, 2015, 102(2): 454-63.

[11] TONG C, MENG Y, LI T, et al. High levels of physical activity are associated with a reduced likelihood of depressive symptoms in postmenopausal women [J]. Women Health, 2023: 1-11.

[12] HO S C, LIANG Z, YU R H, et al. Association of life events and depressive symptoms among early postmenopausal Chinese women in Hong Kong [J]. Menopause, 2017, 24(2): 180-6.

[13] PERSONS J E, ROBINSON J G, CORYELL W H, et al. Longitudinal study of low serum LDL cholesterol and depressive symptom onset in postmenopause [J]. J Clin Psychiatry, 2016, 77(2): 212-20.

[14] PERQUIER F, RYAN J, ANCELIN M-L, et al. Lifetime endogenous reproductive factors and severe depressive symptoms in postmenopausal women: findings from the E3N cohort [J]. Menopause-the Journal of the North American Menopause Society, 2013, 20(11): 1154-63.

[15] LI F, HE F, SUN Q, et al. Reproductive history and risk of depressive symptoms in postmenopausal women: A cross-sectional study in eastern China [J]. J Affect Disord, 2019, 246: 174-81.

[16] JUNG S J, SHIN A, KANG D. Hormone-related factors and post-menopausal onset depression: results from KNHANES (2010-2012) [J]. J Affect Disord, 2015, 175: 176-83.

[17] JUNG S J, SHIN A, KANG D. Menarche age, menopause age and other reproductive factors in association with post-menopausal onset depression: Results from Health Examinees Study (HEXA) [J]. J Affect Disord, 2015, 187: 127-35.

[18] WIE J H, NAM S K, KO H S, et al. The association between abortion experience and postmenopausal suicidal ideation and mental health: Results from the 5th Korean National Health and Nutrition Examination Survey (KNHANES V) [J]. Taiwan J Obstet Gynecol, 2019, 58(1): 153-8.

[19] PARK S, CHOI N-K. Breastfeeding reduces risk of depression later in life in the postmenopausal period: A Korean population-based study [J]. Journal of Affective Disorders, 2019, 248: 13-7.

[20] TSILIGIANNI I G, TYROVOLAS S, BOUNTZIOUKA V, et al. Depressive symptoms in postmenopausal women: results from the MEDIS Study [J]. Women Health, 2014, 54(5): 389-401.

[21] COLANGELO L A, CRAFT L L, OUYANG P, et al. Association of sex hormones and sex hormone-binding globulin with depressive symptoms in postmenopausal women: the Multiethnic Study of Atherosclerosis [J]. Menopause, 2012, 19(8): 877-85.

[22] RYAN J, BURGER H G, SZOEKE C, et al. A prospective study of the association between endogenous hormones and depressive symptoms in postmenopausal women [J]. Menopause-the Journal of the North American Menopause Society, 2009, 16(3): 509-17.
